# Coupling of Charge Regulation and Conformational Equilibria in Linear Weak Polyelectrolytes: Treatment of Long-Range Interactions via Effective Short-Ranged and pH-Dependent Interaction Parameters

**DOI:** 10.3390/polym10080811

**Published:** 2018-07-24

**Authors:** Pablo M. Blanco, Sergio Madurga, Francesc Mas, Josep L. Garcés

**Affiliations:** 1 Physical Chemistry Unit, Department of Materials Science and Physical Chemistry & Research Institute of Theoretical and Computational Chemistry (IQTCUB) of Barcelona University (UB), 08028 Barcelona, Catalonia, Spain; fmas@ub.edu; 2 Department of Chemistry, Technical School of Agricultural Engineering & Agrotecnio of Lleida University (UdL), 25003 Lleida, Catalonia, Spain; jlgarces@quimica.udl.cat

**Keywords:** polyelectrolytes, charge regulation, long-range interactions, Debye–Hückel interactions, transfer matrix, Ising models, semi-grand canonical ensemble, Monte Carlo simulations, conformational equilibria, variational methods

## Abstract

The classical Rotational Isomeric State (RIS) model, originally proposed by Flory, has been used to rationalize a wide range of physicochemical properties of neutral polymers. However, many weak polyelectrolytes of interest are able to regulate their charge depending on the conformational state of the bonds. Recently, it has been shown that the RIS model can be coupled with the Site Binding (SB) model, for which the ionizable sites can adopt two states: protonated or deprotonated. The resulting combined scheme, the SBRIS model, allows for analyzing ionization and conformational equilibria on the same foot. In the present work, this approach is extended to include pH-dependent electrostatic Long-Range (LR) interactions, ubiquitous in weak polyelectrolytes at moderate and low ionic strengths. With this aim, the original LR interactions are taken into account by defining effective Short-Range (SR) and pH-dependent parameters, such as effective microscopic protonation constants and rotational bond energies. The new parameters are systematically calculated using variational methods. The machinery of statistical mechanics for SR interactions, including the powerful and fast transfer matrix methods, can then be applied. The resulting technique, which we will refer to as the Local Effective Interaction Parameters (LEIP) method, is illustrated with a minimal model of a flexible linear polyelectrolyte containing only one type of rotating bond. LEIP reproduces very well the pH dependence of the degree of protonation and bond probabilities obtained by semi-grand canonical Monte Carlo simulations, where LR interactions are explicitly taken into account. The reduction in the computational time in several orders of magnitude suggests that the LEIP technique could be useful in a range of areas involving linear weak polyelectrolytes, allowing direct fitting of the relevant physical parameters to the experimental quantities.

## 1. Introduction

The ionization state of charged macromolecules in solution is regulated by the binding of small ions (protons, metal ions, etc.) present in the backward medium. In particular, acid-basic equilibria in weak polyelectrolytes represent the paradigmatic mechanism of charge regulation due to the ubiquitous presence of proton ions in aqueous solution. These processes are of paramount importance to understand the physicochemical behavior of charged macromolecules in a wide range of situations. Just to mention a few examples, charge regulation plays a fundamental role in receptor–ligand equilibria in biochemical systems [1,2,3,4], supramolecular chemistry [5,6,7], the role of natural organic matter in geochemical cicle of metal ions [8], wastewater treatment [9], stability of colloidal systems [10], advanced coating in material science [11,12,13] or drug delivery [14]. Charge regulation can take place on rigid structures, such as surfaces or nano-particles [15], but in general polyelectrolytes are flexible and conformational and ionization degrees of freedom are strongly coupled. This fact can result in dramatic structural changes in the macromolecule. Classical examples are the helix–coil transitions of poly(peptides) [16], the swelling of poly(methacrylic) acid in a very narrow range of pH [17] or the strong influence of ionization in the folding of proteins [18]. More recently, the importance of the ionization configuration in the conformational properties of intrinsically disordered proteins, whose function-structure relationship still remains a controversial matter, has been recognized [19,20].

**Figure 1:**
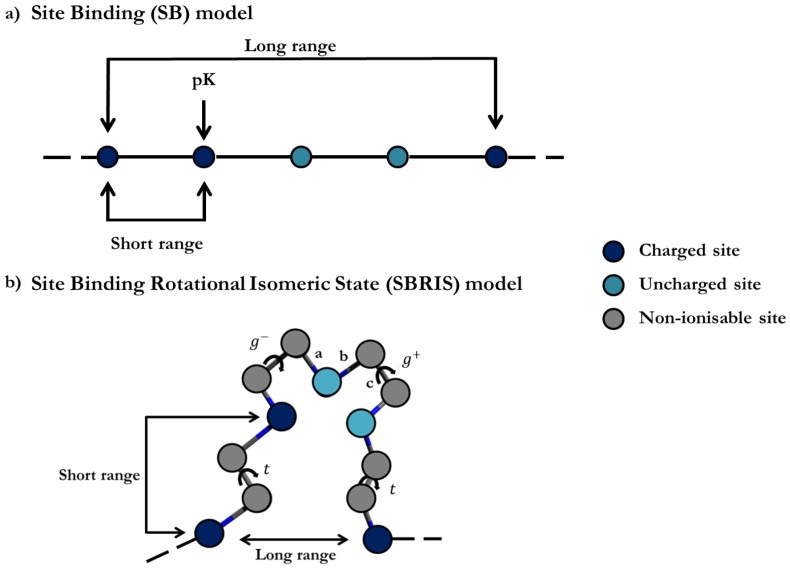
(**a**) Outline of the Site Binding (SB) model for a linear polyelectrolyte represented as a linear chain of ionizable sites. The ionization state of the macromolecule is characterized by a set of variables $s=\left\{s_{i}\right\}$, *i* = 1, ... , *N*, which can adopt two possible values: $s_{i}=1$ if the site *i* is protonated (dark blue circles) and $s_{i}=0$ if it is deprotonated (cyan circles); (**b**) sketch of a linear chain joining ionizable sites by means of rotating bonds as an example of the Site Binding-Rotational Isomeric State (SBRIS) model. Both ionization and conformation degrees of freedom are now taken into account. In the depicted chain, only the bonds type **c** are able to rotate, which can take three possible states of minimum energy, i.e., *trans*, *gauche*+ and *gauche*−.

The understanding of the ionization processes has been mainly based on the so-called Site Binding (SB) model. In this approach, the ionization configuration of the macromolecule is defined as a set of sites which can be in two possible states, i.e., protonated or deprotonated, as outlined in Figure 1a. The free energy is then parametrized by site-specific microscopic protonation constants and interaction energies between sites. Triplet or higher-order interactions among sites can also be considered. Once the system is parametrized, the machinery of statistical mechanics can be used in order to quantify the relevant physical properties such as titration curves, site-specific binding probabilities, macroscopic protonation constants, microscopic protonation enthalpies, site–site binding correlation, etc. [5,15,21,22,23,24,25]. For systems with a small number of sites (N≤20), the necessary thermal averages can be performed by direct enumeration, while, for a large number of sites, Monte Carlo (MC) simulations become necessary [26,27,28,29,30,31,32,33,34,35,36,37].

In the case of linear polyelectrolytes, the transfer matrix method can be used to compute the relevant thermal averages [15]. This powerful and elegant technique was originally designed to solve the classical Ising model of ferromagnets. It is based on the fact that the partition function of a system with *N* + 1 sites can be related in a recursive way to the one of a system with *N* sites. The resulting recursive relationship can be expressed in terms of the transfer matrix, whose elements represent the contributions of the new site to the partition function, for a given state of the preceding site [38,39]. The technique is very versatile and can be generalized to systems composed with repetitive units (spins, bonds or binding sites), which can take in principle more than two states. When applied to the binding of ions to polyelectrolytes, the method can be adapted to include a wide range of phenomena such as triplet interactions between sites [21], chelate complexation of metal ions [23], proton binding to polyampholytes [40,41], protein-DNA binding [42], super-capacitator charging [43] or coupling between ionization and conformational degrees of freedom [44,45,46].

Probably the most productive application of transfer matrices was proposed by Flory in the context of the Rotational Isomeric State (RIS) model [47,48], aiming to compute conformational properties of neutral linear molecules. The RIS model relies on the observation that, although a particular bond can adopt in principle any rotation angle, only those of minimum energy (typically *trans*, *gauche*+ and *gauche*−) are significantly populated. As a consequence, each bond can be regarded as a ‘unit’ of the system adopting three possible states. The corresponding partition function and the necessary thermal averages (bond probabilities, end-to-end distance, radius of gyration, etc.) can be calculated using a proper product of transfer matrices. In recent works [45,46], it has been shown that SB and RIS models can be combined in a unique scheme so that conformational and ionization equilibria can be analyzed on the same foot. It has been shown that all the matricial expressions of RIS can be systematically extended to account for the ionization degrees of freedom. The resulting SBRIS model, outlined in Figure 1b, has been recently applied to the detailed characterization of the conformational and ionization properties of linear poly(ethylene)imine [46].

The main limitation of the transfer matrices used in SB, RIS and SBRIS models is that they can only deal with Short-Range (SR) interactions [49,50]. SR interactions are chemically specific and can produce important correlations between neighbouring sites and bonds. They cannot be modeled by simple continuous force fields (such as van der Waals or Debye–Hückel potentials) [51] but, in exchange, they can be easily implemented in a transfer matrix scheme. For polyelectrolytes, however, this is an important restriction, due to the Long-Range (LR) nature of coulombic interactions, which severely restricts the range of application of the transfer matrix approach. In practice, the possibility of neglecting LR coulombic interactions must be restricted to high ionic strengths, an important limitation specially for polyelectrolytes which become insoluble under such conditions [52,53,54,55].

In a recent paper [56], the SB model has been extended to include LR interactions by introducing a modified free energy involving Local Effective Interaction Parameters (LEIP), which account for the LR interactions in an effective way. The LR force field is thus replaced by a short-ranged effective one. The new local effective parameters, i.e., effective protonation free energies, effective pair interactions and so on, can be systematically calculated by using the Gibbs–Bogoliubov variational principle [39]. The resulting modified free energy converges very fast to the exact free energy. It was found that the correction to the site protonation p*K* (first order correction) is enough to obtain an excellent, exact from a practical point of view, agreement between theory and MC simulations. This previous study, however, was restricted to rigid molecules, and conformational degrees of freedom were not taken into account.

The main goal of the present work is to extend the LEIP method to account for the coupling between charge regulation and conformational equilibria involving LR interactions. In addition to allow much faster computations of ionization/conformational properties (computational times are reduced in orders of magnitude), the methodology here presented adds new physical insight in the interplay of conformational and ionization degrees of freedom in polymeric structures. For instance, the energy of the *gauche* state of a bond will now depend on the pH and the ionic strength, even if such a bond does not hold any ionizable group. The use of the LEIP technique in the SB model is reviewed in Section 2. In Section 3, the technique is generalized in order to include conformational equilibria coupled to LR coulombic interactions represented by the Debye–Hückel potential. In Section 4, semi-grand canonical Monte Carlo simulations are introduced as a tool to test LEIP accuracy when applied to flexible polyelectrolytes. In Section 5, LEIP theoretical results are compared to MC simulations. The new ideas here introduced are illustrated with a minimal model of a flexible linear weak polyelectrolyte containing only one type of rotating bond.

## 2. Simultaneous Treatment of Short- and Long-Range Interactions in Rigid Molecules

The ionization state of a macromolecule with *N* ionizable sites can be characterized by a set of variables $s=\left\{s_{i}\right\}$, *i* = 1,... , *N*, which can adopt two possible values: $s_{i}=1$ if the site *i* is protonated, and $s_{i}=0$ if it is deprotonated. The corresponding reduced free energy can be expressed in terms of the variables $s_{i}$ by means of the so-called cluster expansion [24]

(1) $$\frac{H\left(s\right)}{\ln10}=\sum \mu_{i}s_{i}+\sum_{i>j}\phi_{ij}s_{i}s_{j}+\sum_{i>j>k}\tau_{ijk}s_{i}s_{j}s_{k}+\ldots ,$$

where $\mu_{i}=\mathrm{pH}-pK_{i}=-\log\left(K_{i}a_{H}\right)$ is the reduced chemical potential, which depends on the proton activity, $a_{H}$, and the protonation p*K*-value of the ionizable site *i*, $pK_{i}$; $\phi_{ij}$ represents the interaction energy of the sites *i* and *j*; $\tau_{ijk}$ accounts for possible triplet interactions among sites *i*, *j* and *k*, and so on. The term “reduced” refers to the fact that the chemical potential incorporates both the pH and the protonation p*K*, which simplifies the subsequent expressions. The interaction (or cluster) parameters are expressed in thermal units, i.e., $\beta =1/k_{B}T=1$, and divided by a factor ln10 in order to be compared in the pH scale. Note that the conformation degrees of freedom are omitted in Equation (1), so that the interaction parameters should be understood as proper averages over the conformational states. The mathematical form of these averages is not trivial and expressions for them are given in [45]. Throughout this work, we will assume that a site is charged when it is protonated, i.e., we are dealing with poly-cations. However, the subsequent arguments are also applicable to poly-anions with a suitable change in the protonation variables [15]. The expansion of the free energy (1) usually converges very fast to the exact free energy, and, for most of the cases, the inclusion of triplet interactions is enough to accurately reproduce the measurable quantities, such as the degree of ionization of the individual sites [22]. These can be obtained from $H\left(s\right)$ by means of the semi-grand canonical partition function

(2) $$\Xi =\sum_{s}e^{-H\left(s\right)}.$$

The average degree of protonation of a particular site *i* is related to Ξ as

(3) $$\theta_{i}=\langle s_{i}\rangle =-\frac{\partial \log\Xi }{\partial \mu_{i}}=\frac{1}{\ln10}\frac{\partial \Omega }{\partial \mu_{i}},$$

where Ω=−lnΞ is the thermodynamic potential associated with the semi-grand canonical ensemble. The average number of bound protons is given by

(4) $$\nu =\langle \sum_{i}s_{i}\rangle =\frac{\partial \Omega }{\partial \ln a_{H}}.$$

The correlation of the protonation degrees of two sites *i* and *j*, a quantity which will be used later, can be expressed as

(5) $$h_{ij}=\langle s_{i}s_{j}\rangle =-\frac{1}{{\left(\ln10\right)}^{2}}\frac{\partial \Omega }{\partial \mu_{i}\partial \mu_{j}}.$$

As can be seen, the quantification of all the relevant physical quantities relies in the accurate determination of the partition function Ξ. If the number of sites is small (N≤20), Ξ can be evaluated by direct enumeration of all the possible ionization states. Otherwise, Monte Carlo (MC) simulations must be performed. In some cases, however, methods borrowed from Statistical Mechanics can be used. Among them, probably the most elegant one is the transfer matrix method, consisting of relating the partition function for a system with *N* + 1 sites with that with *N* sites in a recursive way. This method was firstly used in the exact solution of the Ising model of ferromagnets [38,39]. The link between both partition functions is the transfer matrix whose elements are the Boltzmann factors corresponding to the increase in the reduced free energy. For instance, for the linear polyelectrolyte sketched in Figure 1a and assuming only nearest neighbour interactions, the partition function can be expressed as [47]

(6) $$\Xi =qT^{N}p^{T},$$

where T is the transfer matrix

(7) $$T=\left(\begin{array}{cc} 1 & z\\ 1 & z\;u \end{array}\right).$$

$z=Ka_{H}$ represents the reduced activity and ϵ=−logu is the interaction free energy between neighbouring sites. q=(1,0) and p=(1,1) are the initiating and terminating vectors. This would be the simplest use of the transfer matrix.

**Figure 2:**
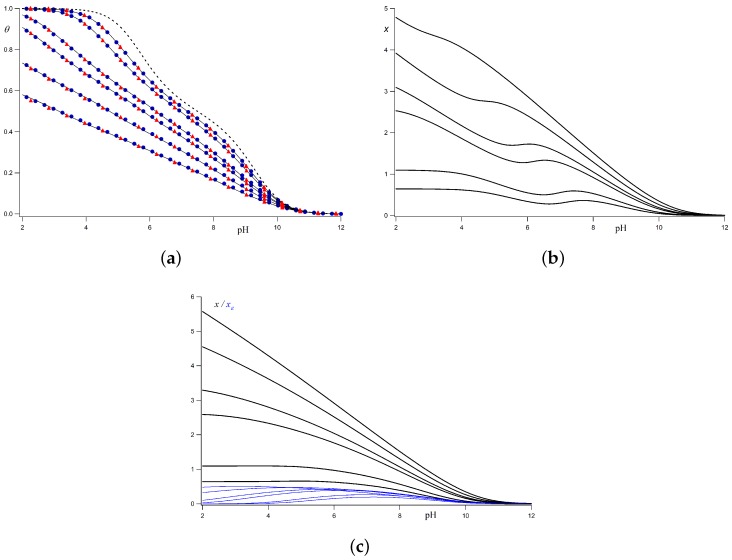
(**a**) Titration curves corresponding to a rigid linear chain with interacting ionizable sites separated by a distance *b* = 0.2 nm obtained using Monte Carlo (MC) simulations (blue circles), Local Effective Interaction Parameters (LEIP) method correcting only the p*K*-value (continuous line) and LEIP method correcting the p*K*-value and the the nearest neighbour interaction energy ϵ (red triangles). The chosen parameters are pK=9 and ϵ=1.5. The Long-Range (LR) interactions are calculated using the Debye–Hückel potential. The dashed line represents the titration curve in the absence of LR interactions. Note that the correction to the p*K*-value is enough to reproduce almost exactly the MC simulations and no significant improvement is obtained in correcting ϵ; (**b**) correction *x* to the p*K*-value using the LEIP method; (**c**) corrections *x* (black lines) and $x_{\epsilon }$(blue lines) to the p*K*-value and the nearest neighbour interaction energy ϵ, respectively. In all the figures, from top to bottom, the ionic strengths are 1 M, 0.5 M, 0.1 M, 0.05M, 0.01 M and 0.001 M.

The main limitation of the transfer matrix methods is that they can be only used when Long-Range (LR) interactions are neglected, since the size of the transfer matrices grows exponentially with the range of the interactions [50]. This is an important limitation of the method when dealing with polyelectrolytes, since it can be only used at high enough ionic strengths, for which the screening is enough to avoid the LR interactions. In a recent paper [56], we introduced a method which allows for including the LR interactions in a very accurate way. In this approach, the full free energy Equation (1) is replaced by a new one involving only Short-Range (SR) interaction parameters, accounting for the LR interactions in an effective way. The resulting formalism deals with both SR and LR interactions simultaneously. The method can be used for any kind of molecular or surface geometry, but it is restricted to rigid structures, so that the conformational degrees of freedom are not explicitly taken into account. Since the main goal of this work is to extend this formalism to flexible molecules and polyelectrolytes, we briefly outline the basic ideas of the method. The details of the derivations are given in Reference [56]. Although the following arguments can be readily generalized to the general form of the free energy Equation (1), let us consider the simplest case of a rigid linear chain with identical sites, such as the one sketched in Figure 1a. For this system, $\mu_{1}=\mu_{2}=\ldots =\mu$ and triplet interactions are omitted, i.e., $\tau_{ijk}=0$. The reduced free energy *H* can be split into two contributions $H=H_{0}\left(x\right)+\Delta H\left(x\right)$ such as

(8) $$\begin{array}{c} \frac{H_{0}}{\ln10}=\left(\mu -x\right)\sum_{i}s_{i}+\epsilon \sum_{i}s_{i}s_{i+1},\\ \frac{\Delta H}{\ln10}=\sum_{j>i+1}\phi_{ij}s_{i}s_{j}+x\sum_{i}s_{i}, \end{array}$$

where *x* is a parameter to be determined. Note that $H_{0}$ corresponds to a reduced free energy containing only nearest neighbour interactions of energy $\epsilon =\phi_{i,i+1}$, which can be exactly solved by using the transfer matrix (7). Now, we can use the Gibbs–Bogoliubov variational principle [39,57]

(9) $$\Omega \leq \tilde{\Omega }=\Omega_{0}\left(x\right)+{\langle \Delta H\left(x\right)\rangle }_{0}$$

to determine the optimal value of *x*, where $\Omega_{0}\left(x\right)=-\ln\Xi_{0}$ and ${\langle \cdots \rangle }_{0}$ represent the free energy and the thermal average corresponding to $H_{0}$, respectively. Minimizing $\tilde{\Omega }$ with respect to *x*, it is found that *x* fulfills the equation [56]

(10) $$x=\frac{d\varphi_{0}/dx}{d\nu_{0}/dx}=\frac{d\varphi_{0}}{d\nu_{0}},$$

where

(11) $$\varphi_{0}={\langle \sum_{j>i+1}\phi_{ij}s_{i}s_{j}\rangle }_{0}=\sum_{j>i+1}\phi_{ij}h_{ij}^{0}$$

is the LR energy averaged over the unperturbed free energy $H_{0}$, whose correlation function $h_{ij}^{0}$, can be exactly evaluated using (5). If the optimal value for *x* is used in the computations, the variational principle (9) implies that all the thermal averages (degree of protonation, correlation functions, etc.) can be obtained replacing the average $\langle \cdots \rangle$ by ${\langle \cdots \rangle }_{0}$, which can be exactly determined since only SR interactions are involved. Equation (10) provides a transparent physical interpretation of *x*: it is the average change in the LR interaction energy when a new proton is bound to the molecule at a given pH-value. As expected, *x* vanishes in the absence of LR interactions and the nearest neighbour interaction model becomes exact. Therefore, *x* can be interpreted as the necessary correction to the reduced chemical potential μ in order to account for the LR interactions but in a local effective way. By the definition of μ=pH−pK, *x* can also be understood as the correction to the site p*K*-value, so that $pK^{\mathrm{eff}}=pK-x$ is the effective p*K*-value, and it represents the extra energetic cost of the site protonation due to the presence of LR interactions. We will refer to this procedure as the Local Effective Interaction Parameters (LEIP) method. It is important to highlight that LEIP, unlike other approaches involving some mean-field approximation (such as the Bragg–Williams approximation in Ising models), includes the correlations via Equation (11), although in an approximate way. This approximation, however, results in being extremely accurate, as can be observed in Figure 2a, where the titration curves corresponding to a rigid linear chain with identical sites are depicted. The chosen parameters are pK=9 and ϵ=1.5. In this model, the LR interactions between distant sites are described by the Debye–Hückel potential

(12) $$\phi_{ij}=\frac{1}{\ln10}\frac{\ell_{B}e^{-\kappa d_{ij}}}{d_{ij}}\;\;\;\;;\;\;\;\;j>i+1,$$

where $\ell_{B}≃0.7$ nm is the Bjerrum length in water at 298 K, $d_{ij}$ is the distance between the sites *i* and *j*, and $\kappa^{-1}\left(\mathrm{nm}\right)=0.304/\sqrt{I\left(M\right)}$ is the Debye length at the ionic strength *I*. For a rigid linear chain, as the one shown in Figure 1, $d_{ij}=\left|j-i\right|b$, where *b* is the separation between consecutive protonating sites. We have plotted the titration curves obtained by Monte Carlo (MC) simulations in the semi-grand canonical ensemble, i.e., at constant pH (blue circles), together with the ones calculated using Equations (10) and (11) (continuous lines) for all the range of ionic strengths and *b* = 0.2 nm. Surprisingly, simulated and calculated curves overlap, so that, for this model, the LEIP solution can be regarded as exact from the practical point of view. The computational cost of LEIP methods is many orders of magnitude lower than that required in MC simulations, allowing the fitting of parameters to experimental titration curves. The correction to the p*K*, *x*, shown in Figure 2b, increases in lowering the pH (i.e., increasing the charge), and in decreasing the ionic strength (lower electrostatic screening), since the energetic cost to protonate a site increases with the macromolecular charge and with the intensity of the LR interactions.

Another advantage of the LEIP method is that it can be systematically improved by selectively correcting other cluster parameters. For instance, one could decide to correct, not only the p*K*-value (pK→pK−x), but also the nearest neighbour interaction energy ($\epsilon \to \epsilon +x_{\epsilon }$). Proceeding in the same way, it can be shown that *x* and $x_{\epsilon }$ fulfill the nonlinear system of equations [56]

(13) $$J\left(\begin{array}{c} x\\ x_{\epsilon } \end{array}\right)=\left(\begin{array}{c} {\left(\frac{\partial \varphi_{0}}{\partial x}\right)}_{x_{\epsilon }}\\ {\left(\frac{\partial \varphi_{0}}{\partial x_{\epsilon }}\right)}_{x} \end{array}\right)\;\;;\;\;J=\left(\begin{array}{cc} {\left(\frac{\partial \nu_{0}}{\partial x}\right)}_{x_{\epsilon }} & {\left(\frac{\partial D_{0}}{\partial x}\right)}_{x_{\epsilon }}\\ {\left(\frac{\partial \nu_{0}}{\partial x_{\epsilon }}\right)}_{x} & {\left(\frac{\partial D_{0}}{\partial x_{\epsilon }}\right)}_{x} \end{array}\right),$$

where $\nu_{0}\left(x,x_{\epsilon }\right)$ and $D_{0}={\langle s_{i}s_{i+1}\rangle }_{0}=h_{12}^{0}\left(x,x_{\epsilon }\right)$ represent the average number of protons and the average number of nearest neighbour interactions, respectively, which can be exactly calculated using $H_{0}$. Solving Equation (13), the correction to the p*K* and ϵ are obtained as functions of the pH. The physical meaning of *x* and $x_{\epsilon }$ becomes transparent if Equation (13) are rewritten in terms of $\nu_{0}$ and $D_{0}$ as independent variables. After some elementary algebra, *x* and $x_{\epsilon }$ adopt the much simpler form

(14) $$x={\left(\frac{\partial \varphi_{0}}{\partial \nu_{0}}\right)}_{D_{0}}\;\;;\;\;\;x_{\epsilon }={\left(\frac{\partial \varphi_{0}}{\partial D_{0}}\right)}_{\nu_{0}}.$$

Equation (14) states that *x* represents the increase in $\varphi_{0}$ for a constant number of interactions $D_{0}$, while $x_{\epsilon }$ can be interpreted as the change in $\varphi_{0}$ in creating a nearest neighbour interaction, keeping constant the number of bound protons $\nu_{0}$. Intuitively, one can guess that $x_{\epsilon }$ is much smaller than *x*, so that the correction to the p*K* is enough to reproduce almost exactly the exact free energy, generating physical properties almost indistinguishable from the MC simulations. In Figure 2a, the titration curves have been recalculated using the correction to ϵ. As expected, no significant improvement is obtained. *x* and $x_{\epsilon }$ as functions of the pH are shown in Figure 2c, where it is clearly observed that $x_{\epsilon }$ is much lower than *x* for all the ionic strengths. Note that the wavy behaviour of *x* in Figure 2b is no longer present in Figure 2c, and seems to be replaced by the contribution $x_{\epsilon }$. Using the same procedure, corrections to higher order interactions, such as triplet or next-nearest neighbour interactions, can be calculated until the desired accuracy is obtained, and expressions of type (14) can be generalized in a straightforward manner. The same treatment leads to very good results for heterogeneous polyelectrolytes and polyampholytes, by correcting the p*K*-values of the different kind of sites ($pK_{i}\to pK_{i}-x_{i}$) [56].

## 3. Coupling of Ionization and Conformational Equilibria

For a linear macromolecule composed by *M* bonds, a particular conformational state is denoted by a set of variables $c=\left\{c_{\alpha }\right\}$, *j* = 1, ..., *M*. The variables $c_{\alpha }$ can adopt several values corresponding to the rotational angles of the bond α. The possible states of the bonds are usually chosen as those of minimum energy, three in the simplest situation: *trans*, *gauche*+ and *gauche*−. The selection of a finite number of rotational states instead of working with the full continuous rotational potential greatly simplifies the statistical mechanics treatment, and constitutes the basis of the Rotational Isomeric State (RIS) model, mainly developed by Flory [47]. In the case of linear polymers, the transfer method can be used to determine the conformational partition function $\Xi_{\mathrm{rot}}$, which can be expressed as

(15) $$\Xi_{\mathrm{rot}}=qU_{1}U_{2}\ldots U_{M-1}U_{M}p^{T},$$

where $U_{\alpha }$ is the transfer matrix corresponding to the bond α. For a symmetric chain, for which the states *gauche*+ and *gauche*− have the same energy and identical bonds, the transfer matrices are of the form

(16) $$U=\left(\begin{array}{ccc} 1 & \sigma & \sigma \\ 1 & \sigma \psi & \sigma \omega \\ 1 & \sigma \omega & \sigma \psi \end{array}\right),$$

where σ, ψ and ω are the Boltzmann factors associated with the conformational energies of the bonds: $-k_{B}T\ln\sigma$ is the free energy of the *gauche* states while ψ (ω) are related to the interaction energies between two consecutive *gauche* states of different (same) orientation. ψ and ω equate one if the rotation of the bonds is independent. $q=\left(1,0,0\right)$ and $p=\left(1,1,1\right)$ are the initiating and terminating vectors. As in the SB model, the necessary thermal averages can be obtained by performing proper derivatives of the partition function. For instance, the average number of bonds in the *gauche* state is given by [47]

(17) $$g=\frac{\partial \ln\Xi_{\mathrm{rot}}}{\partial \ln\sigma }.$$

The RIS model can be generalized in order to take into account the protonation degrees of freedom. If the macromolecule is in a protonation state *s*, the pair $\left(s,c\right)$ defines a *roto-microstate* with reduced free energy

(18) $$F\left(s,c\right)=F_{\mathrm{rot}}\left(c\right)+F_{p}\left(s,c\right).$$

$F_{\mathrm{rot}}\left(c\right)$ is the free energy corresponding to the fully deprotonated state of each conformer, while $F_{p}\left(s,c\right)$ represents the reduced free energy due to the protonation process, which, for a given conformation, can be expressed as

(19) $$\frac{\beta F_{p}\left(s,c\right)}{\ln10}=\sum_{i}\mu_{i}\left(c\right)s_{i}+\sum_{j>i}\phi_{ij}\left(c\right)s_{i}s_{j},$$

where triplet interactions have been neglected. Note that the cluster parameters now depend on the conformational state *c*. The reduced free energy Equations (18) and (19) combines the RIS and the SB model and defines the SBRIS model, which allows for studying conformational and ionization properties on the same foot. In recent publications, the SBRIS model has been used to explain conformational transitions in weak linear polyelectrolytes [45] and in the characterization of ionization/conformational properties of linear poly(ethylenimine) [46]. The probability of a specified roto-microstate is given by

(20) $$p\left(s,c\right)=\frac{e^{-\beta F\left(s,c\right)}}{\Xi_{\mathrm{SBRIS}}},$$

where the SBRIS partition function $\Xi_{\mathrm{SBRIS}}$ is defined as

(21) $$\Xi_{\mathrm{SBRIS}}=\sum_{s,c}e^{-\beta F\left(s,c\right)}.$$

The SBRIS partition function can alternatively be expressed in the fashion

(22) $$\Xi_{\mathrm{SBRIS}}=\sum_{s}\Xi_{\mathrm{rot}}\left(s\right),$$

where $\Xi_{\mathrm{rot}}\left(s\right)$ denotes the rotational partition function for the macromolecule in a ’frozen’ binding configuration $s=\left\{s_{1},s_{2},\cdots ,s_{N}\right\}$. $\Xi_{\mathrm{rot}}\left(s\right)$ can then be calculated as a RIS partition function as in Equation (15), but now decorating the transfer matrices with the suitable binding parameters. The sum over the protonation states can be performed by using proper matricial methods described elsewhere [46]. They are outlined as supplementary information and here we just comment the final results. The SBRIS partition function is obtained by replacing the conformational RIS transfer matrices **U** (Equation (16)) for suitable super-matrices. The rule is that, if a bond is holding at its ends two ionization groups, **U** must be replaced by

(23) $$U\to B=\left(\begin{array}{cc} U & Uz\\ U & Uuz \end{array}\right),$$

where **u** is a diagonal matrix containing the Boltzmann factors corresponding to the short-range interactions

(24) $$u=\left(\begin{array}{ccc} u_{t} & 0 & 0\\ 0 & u_{g} & 0\\ 0 & 0 & u_{g} \end{array}\right).$$

In matrix (24), $-k_{B}T\ln u_{t}$ and $-k_{B}T\ln u_{g}$ represent the short-range interaction energy between two protonated sites separated by a bond in *trans* and *gauche* conformation, respectively. For the bonds which do not hold ionization sites, the substitution is

(25) $$U\to B=\left(\begin{array}{cc} U & 0\\ 0 & U \end{array}\right).$$

The resulting SBRIS partition function reads

(26) $$\Xi_{\mathrm{SBRIS}}=rB_{1}B_{2}\ldots B_{M-1}B_{M}t^{T},$$

where $r=\left(q\;q\right)$ and $t=\left(p\;p\right)$ are the initiating and terminating vectors, respectively. The average number of bound protons and the bond state probabilities can be again obtained by proper derivatives of Equation (26). It can also be shown that matricial expressions for other physical quantities derived in the context of the RIS model can also be generalized to ionizable molecules by performing suitable substitutions by super-matrices [45]. For instance, there are available matricial expressions for the average square distance between two sites of the chain. These expressions are used in this work to estimate the average distance between charged sites and the corresponding LR interaction energy.

**Figure 3:**
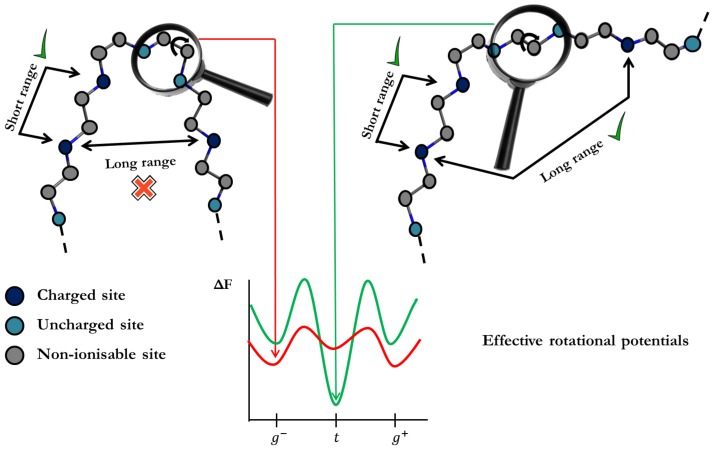
Outline of the Local Effective Interaction Parameters (LEIP) method. LEIP accounts for the Short-Range (SR) interactions exactly, but LR interactions are replaced by effective SR free energies. In the resulting scheme, only SR interactions are present, which considerably simplify the theoretical treatment. The bonds “feel” the presence of the LR interactions in an effective way, which leads to an apparent pH-dependent rotational energy. Other parameters, such as the protonation p*K*-values, also become pH-dependent due to the presence of LR interactions.

As commented on in the preceding section, transfer matrix methods can only be applied if only SR interactions are taken into account. In this work, we propose to use the LEIP technique to include the LR interactions via local parameters, as done in the case of rigid molecules. Now, however, not only the ionization parameters, such as pK→pK−x, but also the conformational parameters must be corrected as outlined in Figure 3. In the simplest case, with only one kind of rotating bonds, the substitution $p\sigma \to p\sigma +x_{\sigma }$ where pσ=−logσ will be necessary. The treatment is almost identical to the one used for rigid molecules. Now, the “unperturbed” free energy is $\Omega_{0}=-\ln\Xi_{\mathrm{SBRIS}}\left(x,x_{\sigma }\right).$ It can be easily shown that the corrections *x* for the p*K* and $x_{\sigma }$ for pσ fulfill equivalent equations to (13)

(27) $$J\left(\begin{array}{c} x\\ x_{\sigma } \end{array}\right)=\left(\begin{array}{c} {\left(\frac{\partial \varphi_{0}}{\partial x}\right)}_{x_{\sigma }}\\ {\left(\frac{\partial \varphi_{0}}{\partial x_{\sigma }}\right)}_{x} \end{array}\right)\;\;;\;\;J=\left(\begin{array}{cc} {\left(\frac{\partial \nu_{0}}{\partial x}\right)}_{x_{\sigma }} & {\left(\frac{\partial g_{0}}{\partial x}\right)}_{x_{\sigma }}\\ {\left(\frac{\partial \nu_{0}}{\partial x_{\sigma }}\right)}_{x} & {\left(\frac{\partial g_{0}}{\partial x_{\sigma }}\right)}_{x} \end{array}\right),$$

where $\nu_{0}\left(x,x_{\sigma }\right)$ represents the average number of bound protons (Equation (4)) and $g_{0}\left(x,x_{\sigma }\right)$ the average number of bonds in the *gauche* state (Equation (17)), calculated using the unperturbed free energy. If we use $\nu_{0}$ and $g_{0}$ as independent variables, instead of *x* and $x_{\sigma }$, Equation (27) can be rewritten in a similar fashion as Equation (14)

(28) $$x={\left(\frac{\partial \varphi_{0}}{\partial \nu_{0}}\right)}_{g_{0}}\;\;;\;\;\;x_{\sigma }={\left(\frac{\partial \varphi_{0}}{\partial g_{0}}\right)}_{\nu_{0}},$$

which essentially tell us that *x* represents the average change in $\varphi_{0}$ when a new proton is bound (keeping constant the number of bonds in *gauche*) while $x_{\sigma }$ is the the average change in $\varphi_{0}$ when a bond is brought to its *gauche* state (keeping constant the number of bound protons). Note that the LEIP method always leads to expressions for the interaction corrections of the same type of Equations (14) and (28).

As in the previous section, LR interactions are described by the Debye–Hückel potential, although the method could in principle be applied to other kind of interactions such as van der Waals interactions. Moreover, by including convenient “hard core” terms in the interaction potentials, the excluded volume effect could in principle be taken into account. The study of this effect, however, is not trivial and it is out of the scope of this work. Unlike rigid molecules, for flexible molecules, the average LR interaction energy $\varphi_{0}$ for the unperturbed free energy can only be approximately calculated. In this work, as a first approximation, we assume that

(29) $$\varphi_{0}={\langle \sum_{ij}\phi \left(d_{ij}\right)s_{i}s_{j}\rangle }_{0}≃\sum_{ij}\phi \left(\sqrt{{\langle d_{ij}^{2}\rangle }_{0}}\right){\langle s_{i}s_{j}\rangle }_{0}.$$

This approximation could in principle be improved by using higher order moments of $d_{ij}$. Matricial expressions for ${\langle d_{ij}^{2}\rangle }_{0}$ and higher moments where derived by Flory and Jernigan [47,58] for neutral chains. Here, these expressions are modified in order to account for the protonation degrees of freedom. An outline of the derivations is provided as supplementary information.

## 4. Monte Carlo Simulations

**Figure 4:**
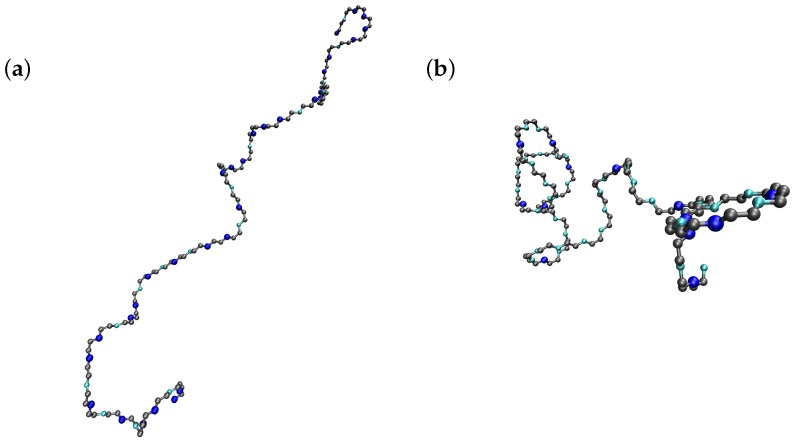
Two snapshots of Monte Carlo simulations with pK=9, $\epsilon_{t}=1$, $u_{g}=0$, σ=10 and pH=4 (**a**) and pH=8 (**b**). Note that elongated conformations are promoted at low pH as a consequence of polyelectrolyte global charge increase.

In order to estimate the accuracy of the LEIP method when applied to flexible polyelectrolytes, we compare the theoretical values with those resulting from MC simulations. Two main MC techniques have been previously proposed: the Reaction Ensemble approach, for which the pH is a calculated quantity [59,60], and the constant pH method, corresponding to the semi-grand canonical ensemble [33,61,62,63]. Since the control variable in the LEIP method is the pH-value, as indicated by the reduced free energies in Equations (1) and (18), the constant pH method has been chosen here. In previous studies about polyelectrolyte ionization properties, both Reaction Ensemble and constant pH methods have been coupled to Molecular Dynamics schemes in order to deal with explicit ions. In this work, free protons, co- and counter-ions are not explicit in the simulations and the screening effects are taken into account via the Debye length parameter, $\kappa^{‒1}$. The MC code generalises the one previously used in the computation of conformational and ionization properties of linear poly(ethylenimine) [46]. The polyelectrolyte is modeled as a linear chain with rigid bond lengths and angles. Bonds can adopt one of the three states of minimum energy (*trans*, *gauche*+ or *gauche*−). Each change of a bond state implies a 120° rotation of its dihedral angle and the recalculation of distances among the sites situated before and after the rotating bond. The linear chain is composed of interacting nodes which can correspond to inert or protonating groups. In Figure 4, two snapshots of Monte Carlo simulations at ionic strength 0.001 M and two pH-values (four in Figure 4a and eight in Figure 4b) are presented. As in Figure 1 and Figure 3, the ionizable sites are depicted in blue (dark blue if they are protonated and cyan otherwise). It can be observed that a decrease in the pH-value promotes the elongation of the chain, and the consequent reduction of the electrostatic repulsion, by increasing the number of bonds in the *trans* conformations.

In the MC simulations, the free energy of the system is divided into SR and LR terms

(30) $$F\left(s,c\right)=F_{\mathrm{SR}}+F_{\mathrm{LR}}+\sum_{i}\left(\mathrm{pH}-pK_{i}\right)s_{i}.$$

The SR term is computed using SBRIS free energy (Equation (18)) which involves the energies present in the transfer matrices (σ, ψ, ω, $u_{t}$ and $u_{g}$), while the LR contribution is calculated using the Debye–Hückel potential (Equation (12)). If $F_{LR}$ is set to zero, the obtained results coincide, within the numerical error, with those obtained using the transfer matrix method. This was one of the tests used to check the reliability of the Monte Carlo code. A Metropolis algorithm [15,27] is used to generate roto-microstates at constant pH in a chain with 50 ionizable sites (i.e., 148 nodes or 147 bonds). In each new MC configuration, the polyelectrolyte can change either (i) the conformational state of a rotating bond or (ii) the ionization state of a binding site, with trial probabilities of 0.999 and 0.001, respectively. These trial probabilities allow us to obtain a good equilibration of the conformational structure for each ionization state and the system does not become trapped in local minima. The probability to accept a new configuration is obtained by computing the free energy difference (ΔF(s,c)) between trial and actual conformations. When the state of the bond α is changed, the following free energy differences must be calculated: (i) the conformational energy of bond α and its interaction with bonds α±1 (corresponding to the parameters σ, ψ and ω); (ii) the electrostatic SR interaction between the two sites bound to α when they are charged (corresponding to $u_{t}$ and $u_{g}$, which depend on the new conformation of α ); and (iii) the change in the LR Debye–Hückel interaction among sites before and after α, which involves the recalculation of the distances between the charged sites. On the other hand, a change in the ionization state of a site $s_{i}$ implies to recalculate: (i) the reduced chemical potential of the site *i* by an amount $\Delta \mu_{i}=\left(\mathrm{pH}-pK_{i}\right)\Delta n$, where Δn=±1 is the variation in the number of protons; (ii) the SR repulsive interaction between $s_{i}$ and $s_{i\pm 1}$; and (iii) the LR Debye–Hückel interactions between the trial protonating site and the rest of ionized sites. Once ΔF(s,c) is computed, the new configuration is always accepted if ΔF(s,c)<0 and accepted with a probability exp(−βΔF(s,c)) if ΔF(s,c)>0. The values presented are the average over eight different MC simulations. Each MC simulation has been equilibrated in the first $5\times 10^{7}$ configurations and the thermal averages have been computed in the following $4.5\times 10^{8}$ realizations. The simulations were performed using a parallel code developed in C++ on a 126 CPU cluster. For each pH and ionic strength (one point of the curves), typical jobs were run using 8 CPUs during 1 to 2 h.

## 5. Results and Discussion

As a model system, we use the linear polyelectrolyte outlined in Figure 1b, with protonating sites situated every three chain positions. Only **c** bonds are allowed to rotate and they can adopt the three states of minimum energy, i.e., *trans*, *gauche*+ or *gauche*−. The conformation of **c** bonds determines the intensity of the SR interactions between neighbouring protonated sites. The rest of bonds (**a** and **b** in the figure) are forced to be in the *trans* state. The energy of the *gauche* state of the **c** bonds is denoted as pσ=−logσ. The **c** bonds are assumed to rotate independently when the macromolecule is uncharged (ω=ψ=1 in Equation (16)). The protonating sites are considered to be identical with the same protonation p*K*-value. The interactions between protonated sites are characterized by the energies $\epsilon_{t}=-\log u_{t}$ (when the bond **c** is in *trans* state) and $\epsilon_{g}=-\log u_{g}$ (when the bond **c** is in *gauche* state). In the computations, the used values of the bond length and the bond angle are 0.2 nm and 120°, respectively. This model can be regarded as a minimal model of a flexible polyelectrolyte, with only four energetic parameters involved (σ, $\epsilon_{t}$, $\epsilon_{g}$ and p*K*), and it is here used to illustrate the application of the LEIP method to the analysis of the interplay of conformational and protonation degrees of freedom.

**Figure 5:**
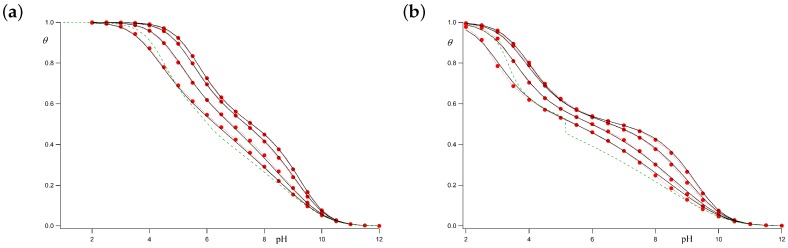
Titrations curve for the model polyelectrolyte depicted in Figure 2 with pK=9, $\epsilon_{t}=1$, $u_{g}=0$ (i.e., $\epsilon_{g}\to \infty$) and σ=1 (**a**) ; σ=10 (**b**). The chosen ionic strengths are, from top to bottom, 1 M, 0.1 M, 0.01 M and 0.001 M. Black continuous lines represent calculations using the LEIP method in which two effective short-range parameters (pK and pσ) has been corrected. Red circles depict the MC values. The green dashed line corresponds to the LEIP values for I=0.001 M when only the pK-value is corrected, while pσ is kept constant.

Let us firstly consider the case for which **c** bonds can freely rotate when the adjacent sites are deprotonated (i.e., σ=1). When both sites are charged, however, the very strong SR repulsion hinders the *gauche* conformation, so that we take $u_{g}=0$ (i.e., $\epsilon_{g}\to \infty$). The resulting titration curves are shown in Figure 5a for ionic strengths ranging from 1 M to 0.001 M. The chosen parameters are pK=9 and $\epsilon_{t}=1$. The black continuous lines represent the average protonation degree θ calculated using the LEIP method correcting both the p*K*-value (pK→pK−x) and the conformational energy of **c** bonds ($p\sigma \to p\sigma +x_{\sigma }$), while the red circles represent the results of the MC simulations. It is observed that the LEIP method reproduces very accurately the MC simulations for all the range of pH-values and ionic strengths. The dashed line depicts the values provided by LEIP for *I* = 0.001 M if only the p*K*-value is corrected, while σ remains constant. Although relative good prediction of the MC simulations is obtained, the quality of the titration curve clearly improves if σ is corrected. This means that the rotational energy of the **c** bonds is affected by LR interactions even if their pendant sites are not charged, as a result of the tendency of the chain to separate the rest of charged groups. Actually, the system behaves as if **c** bonds “feel” the LR interactions in an effective way. This effect is more remarkable in the subsequent case.

Let us check the accuracy of LEIP method when the *gauche* states of the **c** bonds are favored, for instance, because of the existence of hydrogen bond, which means that σ>1. When the adjacent sites are both protonated, on the contrary, the electrostatic repulsion is so strong that the *gauche* states are forbidden ($u_{g}=0$). In this case, the conformational propensity changes when the ionization state of the sites change. Figure 5b compares the titration curves obtained using LEIP correcting p*K* and pσ and MC simulations for pK=9, $\epsilon_{t}=1$ and σ=10. As can be observed, LEIP method and MC simulations yield to almost identical titration curves. In this case, however, the correction of pσ becomes compulsory. If only pK is corrected, the titration curve obtained by LEIP at 0.001 M (green dashed line) exhibits a phase transition-like behaviour at pH≈5. This is an artifact resulting from the impossibility to explain the complex interplay of charge regulation and conformational transition without taking into account the influence of LR interactions in the effective energy of the *gauche* state.

**Figure 6:**
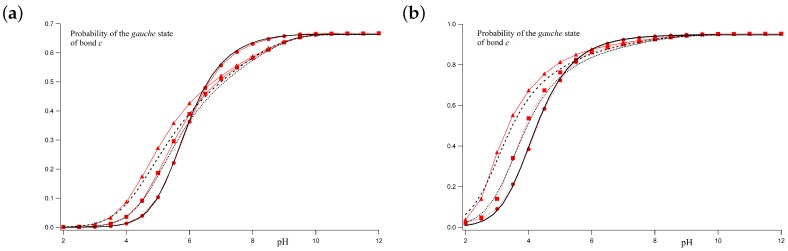
*Gauche* state probabilities of bond **c** versus the pH-value computed by means of MC simulations (red markers) and using the LEIP method (black lines) for ionic strengths: 1 M (circles and continuous line), 0.01 M (squares and dotted line) and 0.001 M (triangles and dashed lines). The chosen parameters are pK=9, $\epsilon_{t}=1$, $u_{g}=0$ and σ=1 (**a**); σ=10 (**b**).

For the two cases commented above, the *gauche* state probabilities versus the pH are shown in Figure 6a,b. Markers correspond to MC simulations while black lines represent the theoretical values at ionic strengths 1 M, 0.01 M and 0.001 M, for pK=9, $\epsilon_{t}=1$ and $u_{g}=0$. Figure 6a corresponds to the case with σ=1. A good correspondence between simulated and theoretical profiles is obtained for all the ionic strengths. Since at low pH-values, the polyelectrolyte is almost fully protonated, the *gauche* state probability tends to zero because of the high electrostatic repulsion between the nearest charged sites in the *gauche* position ($u_{g}=0$). On the other hand, at high pH-values, the macromolecule is completely uncharged and the **c** bonds are freely to rotate. As a result, the probability of the two *gauche* conformers tends to 2/3. For *I* = 1 M, the LR interactions can be neglected and total correspondence between simulated and calculated values is found. At higher ionic strengths (0.01 M and 0.001 M), for which the Debye–Hückel potential is not screened enough, some small differences arise. However, still now, good agreement between the LEIP method and MC simulations is observed.

Figure 6b corresponds to the case with σ=10. Simulated and theoretical probabilities are also in good agreement. Again, the *gauche* state probability tends to zero for low pH-values, while, at high pH-values, the population of the *gauche* conformer is 2σ/(2σ+1)=0.95. A continuous transition from *gauche* to *trans* conformations as the pH decreases is observed. This transition is sharper than in the previous case. Note that, from the LEIP point of view, this transition occurs because of a double effect. On the one hand, there is the charging process, so that two adjacent sites tend to minimize the repulsion when the bonds adopt the *trans* conformation. This effect is present even when LR interactions are not present. On the other hand, the effective *gauche* state energy is increasing due to the average effect of the LR interactions ($p\sigma \to p\sigma +x_{\sigma }$). Both effects are important to correctly reproduce the MC simulations. Otherwise, the lack of flexibility in the conformational energy leads to the spurious phase transition observed in Figure 5b (green dashed line).

**Figure 7:**
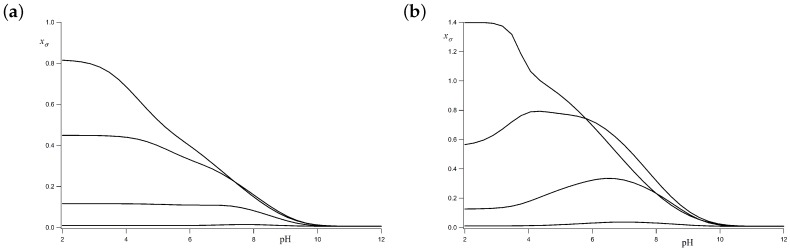
Correction to the *gauche* state energy *$x_{\sigma }$ versus* the pH for pK=9, $\epsilon_{t}=1$, $u_{g}=0$ and σ=1 (**a**); σ=10 (**b**). From the bottom to the top, the ionic strengths are 1 M, 0.1 M, 0.01 M and 0.001 M. $x_{\sigma }$ represents the average effective energy “felt” by **c** bonds as a result of the LR interactions.

Figure 7 shows the LEIP method correction $x_{\sigma }$ to the bond conformational energy ($p\sigma \to p\sigma +x_{\sigma }$) for σ=1 (Figure 7a) and σ=10 (Figure 7b). In both cases, it is observed that $x_{\sigma }$ tends to zero at high pH-values, since the molecule is uncharged and no LR interactions are present, so no correction is necessary. As a general tendency, $x_{\sigma }$ tends to increase as the pH decreases due to the charging process and the corresponding increase in the LR interactions. This effect is larger at low values of the ionic strength since the Debye–Hückel potential is less screened. For the case σ=10, a wavy behaviour is observed for ionic strengths 0.1 M and 0.01 M and $x_{\sigma }$ exhibits a smooth maximum at pH≃4, which coincides with the pH regime where the *trans* to *gauche* transition is sharper. This fact could be due to correlations between the rotation of neighbouring bonds or because part of the correction is effectively included in the p*K*-correction *x*. Further analysis would probably be necessary in order to clarify this point.

**Figure 8:**
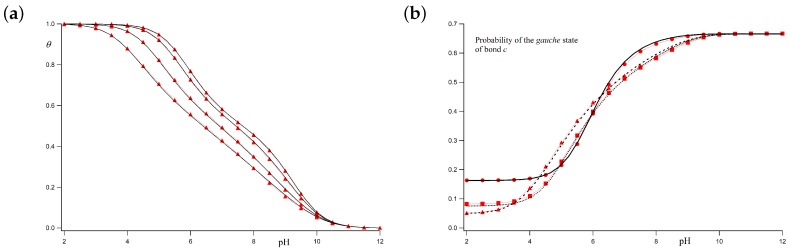
Titration curves (**a**) and *gauche* state probabilities (**b**) obtained using the LEIP method (black lines) and MC simulations (red markers). The chosen parameters are pK=9, $\epsilon_{t}=1$, $\epsilon_{g}=2$ and σ=1. (**a**) the ionic strength are, from top to bottom, 1 M, 0.1 M, 0.01 M and 0.001 M; (**b**) three different ionic strengths are shown: I=1 M (circles and continuous line), 0.01 M (squares and dotted line) and 0.001 M (triangles and dashed line).

In the two cases discussed above, we have taken $u_{g}=0$, which means that bonds between two protonated sites cannot be in the *gauche* state. Let us now relax this condition and take a finite value for $u_{g}$, so that the electrostatic interaction between two charged sites in *gauche* is not forbidden but only penalized. LEIP predictions (black lines) and MC simulations (red markers) are plotted in Figure 8. Figure 8a shows the computed titration curves with $\epsilon_{g}=2$ at ionic strengths ranging, from top to bottom, from 1 M to 0.001 M. Excellent agreement between the theoretical predictions and simulations is obtained for all the ionic strengths, so that the relaxation of the condition $u_{g}=0$ does not seem to affect the accuracy of the LEIP approach. *Gauche* state probabilities versus pH at three ionic strengths are depicted in Figure 8b: 1 M (circles and continuous line), 0.01 M (squares and dotted line) and 0.001 M (triangles and dashed line). As expected, even at low pH-values, some bonds can remain in the *gauche* state due to the finite value of $u_{g}$. Despite the complexity of the obtained profiles for this case, LEIP is able to accurately reproduce the MC simulations.

## 6. Conclusions

The ionization and conformational properties of polyelectrolytes are determined by a combination of Short-Range (SR) and Long-Range (LR) interactions between bonds and ionizable sites. In particular, electrostatic LR interactions can only be neglected at high enough ionic strengths, which is an important limitation for many macromolecular systems of interest. The present work explores the possibility of defining local, short-ranged, interaction parameters which are corrected to account for the LR interactions in an effective way. The new parameters are systematically calculated by using variational methods and equations for them are provided. The resulting approach, the Local Effective Interaction Parameters (LEIP) method, was firstly developed to study the binding properties of rigid polyelectrolytes. In this paper, these ideas are extended to flexible polyelectrolytes, for which conformational and ionization equilibria (charge regulation) are strongly coupled. With this aim, LEIP is combined with the Site Binding Rotational Isomeric State (SBRIS) model in order to deal simultaneously with conformational and protonation degrees of freedom for the full range of ionic strengths.

The LEIP method is illustrated by using a model of a linear symmetric polyelectrolyte containing protonating sites situated regularly along the polymer backbone. The charged sites interact by means of the Debye–Hückel potential, which accounts for the electrostatic screening in an average way, while excluded volume effects are neglected. The bonds linking the ionizable sites can be in three possible states, i.e., *trans*, *gauche*+ and *gauche*−. This model with only four relevant parameters (protonation p*K*-value, *gauche* state energy and SR electrostatic interactions between neighbouring sites through bonds in *trans* and *gauche* states) can be regarded as a minimal model of a flexible polyelectrolyte where conformational and binding equilibria are strongly coupled. The LEIP method is applied to correct both the protonation p*K*-values and the *gauche* state energy. As a result, local pH dependent rotational potentials are obtained. The correction to the *gauche* energy represents the contribution of the LR interactions in rotating a bond to its *gauche* state.

The degree of protonation and the *gauche* state probabilities obtained using the LEIP method are compared with those computed using semi-grand canonical Monte Carlo (MC) simulations. In all of the studied cases, the agreement between LEIP and MC simulations is excellent. The computational cost, however, is orders of magnitude lower in the LEIP method. This fact allows using LEIP to directly fit parameters to experimental information. The LEIP method could also represent a complementary tool to the study of other aspects of the polyelectrolyte physical chemistry, such as the dependence of the molecular size on the pH, the influence of excluded volume interactions, the presence of attractive hydrophobic interactions or the competitive binding of metal ions. The clarification of these points, which have not been the subject of the present study, would be desirable in order to extend the applicability of the LEIP method. We think that the ideas presented here could be useful in the design of pH-dependent force fields based on experimental ionization and conformational properties.

## Supplementary Materials

General equations for SBRIS partition function and mean square distance between sites, necessary in the computations of this work, are provided as supplementary information (can be available online at http://www.mdpi.com/2073-4360/10/8/811/s1). The transfer matrices corresponding to the model polyelectrolyte used here are also reported.

## Abbreviations

The following abbreviations are used in this manuscript:

LEIP	Local Effective Interaction Parameters
LR	Long-Range
MC	Monte Carlo
RIS	Rotational Isomeric State
SB	Site Binding
SBRIS	Site Binding Rotational Isomeric State
SR	Short-Range
