# Supplementary material for: Coupling of Charge Regulation and Conformational Equilibria in Linear Weak Polyelectrolytes: Treatment of Long-Range Interactions via Effective Short-Ranged and pH-Dependent Interaction Parameters
Source: Polymers (Basel). 2018 Jul 24;10(8):811. doi: 10.3390/polym10080811 (PMC6403780; doi:10.3390/polym10080811)
Supplement: Supplementary file 1 [file polymers-10-00811-s001.pdf]

Supplementary information to "Coupling of charge regulation and conformational equilibria in linear weak polyelectrolytes: treatment of long range interactions *via* effective short-ranged and pH-dependent interaction parameters"

July 23, 2018

1 Matricial expression for the SBRIS partition function for a linear polyelectrolyte

Let us express the SBRIS partition function as

$$\Xi_{\mathrm{SBRIS}} = \sum_{s} \Xi_{\mathrm{rot}}\left(s\right) \tag{1}$$

where $\Xi_{\mathrm{rot}}\left(s\right)$ is the partition function for the molecule in a specific binding state $s = \{s_1, s_2, \cdots, s_N\}$. For simplicity, let us suppose that all the N-1 bonds hold two protonating sites at their ends.

The partition function for every 'frozen' binding configuration can then be expressed as a the RIS partition function but decorating the transfer matrices with suitable binding parameters. $\Xi_{\mathrm{rot}}\left(s\right)$ adopts the form [1, 2, 3]

$$\Xi_{\mathrm{rot}}\left(s\right) = \mathbf{p} \prod_{\mathrm{i}=1}^{N-1} \mathbf{U}_i{}^{s_i s_{i+1}} \mathbf{q}^{\mathbf{T}} \tag{2}$$

In the simplest case $\mathbf{U}_i^{00} = \mathbf{U}_i^{10}$; $\mathbf{U}_i^{01} = z \cdot \mathbf{U}_i$ and $\mathbf{U}_{\mathrm{c}}^{11} = z \cdot \mathbf{U}_i \mathbf{u}_i$ where $\mathbf{U}_i$ are the transfer matrices typical of the RIS model for a symmetric polymer

$$\mathbf{U_i} = \begin{pmatrix} 1 & \sigma & \sigma \\ 1 & \sigma\psi & \sigma\omega \\ 1 & \sigma\omega & \sigma\psi \end{pmatrix}_i \tag{3}$$

where z is the reduced activity of the site and $\mathbf{u}$ is a diagonal matrix containing the Boltzmann factors corresponding to the short range interactions between

charged sites

$$\mathbf{u}_i = \begin{pmatrix} u_\mathrm{t} & 0 & 0 \\ 0 & u_\mathrm{g} & 0 \\ 0 & 0 & u_\mathrm{g} \end{pmatrix}_i \tag{4}$$

$-k_\mathrm{B}T \ln u_\mathrm{t}$ and $-k_\mathrm{B}T \ln u_\mathrm{g}$ represent the short range interaction energy between two sites separated by a bond in *trans* and *gauche* conformation, respectively. The next step is to calculate the sum in Eqn. (1), which can be done using the identity

$$\sum_s \left(\prod_{i=1}^{N-1} \mathbf{U}^{s_i s_{i+1}} \right) = (\mathbf{E}\,\mathbf{E}) \prod_{i=1}^{N-1} \begin{pmatrix} \mathbf{U}_i^{00} & \mathbf{U}_i^{01} \\ \mathbf{U}_i^{10} & \mathbf{U}_i^{11} \end{pmatrix} \begin{pmatrix} \mathbf{E} \\ \mathbf{E} \end{pmatrix} \tag{5}$$

where $\mathbf{E}$ is the 3×3 identity matrix. Combining Eqns. (5), (1) and (2), we obtain the SBRIS partition function.

$$\Xi_\mathrm{SBRIS} = \mathbf{s} \prod_{i=1}^{N-1} \begin{pmatrix} \mathbf{U}_i^{00} & \mathbf{U}_i^{01} \\ \mathbf{U}_i^{10} & \mathbf{U}_i^{11} \end{pmatrix} \mathbf{t}^\mathbf{T} \tag{6}$$

where $\mathbf{s} = (\mathbf{p}\,\mathbf{p})$ and $\mathbf{t} = (\mathbf{q}\,\mathbf{q})$. Note that the SBRIS partition function is obtained from the RIS partition function by replacing

$$\mathbf{U} \rightarrow \begin{pmatrix} \mathbf{U} & \mathbf{U}z \\ \mathbf{U} & \mathbf{U}\mathbf{u}z \end{pmatrix} \;;\; \mathbf{p} \rightarrow (\mathbf{p}\,\mathbf{p}) \;;\; \mathbf{q} \rightarrow (\mathbf{q}\,\mathbf{q}) \tag{7}$$

It can be easily shown that if some of the matrices in Eqn. (2) does not depend on any index s_i, the proper substitution is

$$\mathbf{U} \rightarrow \begin{pmatrix} \mathbf{U} & \mathbf{0} \\ \mathbf{0} & \mathbf{U} \end{pmatrix} \tag{8}$$

which can be necessary for the transfer matrices corresponding to bonds which do not hold any ionizable site.

2 Calculation of the mean square distance between two nodes of the chain

The matrix summation trick used above can also be applied to calculate other physical quantities which become pH-dependent in the SBRIS approach. Let $f(c,s)$ be any quantity and $\langle f(c,s) \rangle$ its SBRIS thermal average. Then

$$\langle f(s,c) \rangle = \sum_{s,c} f(s,c) \frac{e^{-\beta F(s,c)}}{\Xi_\mathrm{SBRIS}} = \sum_s \pi(s) \left(\sum_c \frac{f(s,c) e^{-\beta F(s,c)}}{\Xi_\mathrm{rot}(s)} \right) = \sum_s \pi(s) \, \langle f(c,s) \rangle_c \tag{9}$$

where

$$\pi(s) = \frac{\Xi_\mathrm{rot}(s)}{\Xi_\mathrm{SBRIS}} \tag{10}$$

represents the probability of a protonation state s and $\langle f(c,s)\rangle_c$ is the average for that fixed protonation state. Note now that the quantity between brackets in Eqn. (9) represents a typical RIS average which can be calculated using the (conveniently decorated) transfer matrices $\mathbf{U}_i{}^{s_i s_{i+1}}$. In particular, matricial expressions for the mean square distance between two nodes, k and l, of a linear chain are available [1, 4]. For a fixed ionization state, the result is

$$\left\langle d_{kl}^2 \right\rangle(s) = \frac{2}{\Xi_{\mathrm{rot}}(s)} \mathbf{p} \prod_{i=1}^{k} \mathbf{U}_{\mathrm{i}}{}^{s_i s_{i+1}} \left[\mathbf{E}\,\mathbf{0}\,\mathbf{0}\,\mathbf{0}\,\mathbf{0}\right] \prod_{r=k+1}^{l} \mathbf{G}_{\mathrm{r}}{}^{s_i s_{i+1}} \begin{bmatrix} \mathbf{0} \\ \mathbf{0} \\ \mathbf{0} \\ \mathbf{0} \\ \mathbf{E} \end{bmatrix} \prod_{j=l+1}^{N-1} \mathbf{U}_{\mathrm{j}}{}^{s_j s_{j+1}} \mathbf{q} \tag{11}$$

where the matrices $\mathbf{G}$ are proper super-matrices which can be expressed in terms of the transfer matrices $\mathbf{U}_{\mathrm{i}}{}^{s_i s_{i+1}}$, the translation matrices and the bond vectors. The details and derivations are given in the Chapter 4 of the classical Flory's book [1] (see Eqn. 35). Introducing (11) in (9) and using the summation (5), $\left\langle d_{kl}^2 \right\rangle$ is calculated as a function of the pH.

3 Minimal symmetric polyelectrolyte model

The model used in this work to illustrate the LEIP method consists of a polyelectrolyte like the one in Fig. 2b. In this molecule the ionizable sites are separated by three bonds **a**, **b** and **c**. The structure is very similar to the one of poly(oxyethylene) and the corresponding transfer matrices are given can be found in [1, 3]

$$\mathbf{U}_{\mathrm{a}} = \begin{pmatrix} 1 & \sigma_a & \sigma_a \\ 1 & \sigma_a\psi & \sigma_a\omega \\ 1 & \sigma_a\omega & \sigma_a\psi \end{pmatrix} ;\ \mathbf{U}_{\mathrm{b}} = \begin{pmatrix} 1 & \sigma_a & \sigma_a \\ 1 & \sigma_a\psi' & \sigma_a\omega' \\ 1 & \sigma_a\omega' & \sigma_a\psi' \end{pmatrix} ;\ \mathbf{U}_{\mathrm{c}} = \begin{pmatrix} 1 & \sigma & \sigma \\ 1 & \sigma\psi & \sigma\omega \\ 1 & \sigma\omega & \sigma\psi \end{pmatrix}$$

The conformational model here used can be obtained by taking $\sigma_a = 0$, which assures that the **a** and **b** bonds are always in the *trans* state, and $\omega = \psi = \omega' = \psi' = 1$, which means that the bonds of the deprotonated molecule are independent.

References

[1] Flory, P. L. *Statistical Mechanics of Chain Molecules*; John Wiley: New York, **1969**

[2] Garces J. L.; Koper G. J. M.; Borkovec M. *J. Phys. Chem. B* **2006**, *110*, 10937-10950

[3] Garcés J. L.; Madurga S.; Borkovec M. *Phys. Chem. Chem. Phys.*, **2014**, 16, 4626-4638

[4] Flory P. J.; Jernigan R. L. *J. Chem. Phys.* **1965**, 42, 3509-3519
